# Mortality and malformation effects of acute vanadium (V) exposure on the African clawed frog (*Xenopus laevis*) embryos

**DOI:** 10.1007/s11356-023-26196-x

**Published:** 2023-03-10

**Authors:** Simone Dahms-Verster, Amina Nel, Johan H. J. van Vuren, Richard Greenfield

**Affiliations:** Department of Zoology, University of Johannesburg, Cnr. Kingsway and University Rd, Auckland Park, Johannesburg, 2092 Gauteng South Africa

**Keywords:** FETAX, Amphibians, Vanadium pentoxide, Teratogen, LC50, EC50

## Abstract

Vanadium (V) is a transition metal that is found in low concentrations in aquatic ecosystems. These levels increase due to anthropogenic activities. The mortality and teratogenicity effects of V remain unexplored in amphibian species. To address this gap in the knowledge base, a standard Frog Embryo Teratogenic Index – *Xenopus* (FETAX) assessment was conducted. Vanadium pentoxide (V_2_O_5_) was chosen for its known toxicity in other aquatic biota and its solubility in water. A range-finding test was conducted in two different mediums, V_2_O_5_ in distilled water (VDH2O) and V_2_O_5_ in FETAX medium (VMED), to determine concentration ranges where effects occurred. Thereafter, definitive tests were conducted using two separate breeding pairs, with two replicate dishes per concentration containing 15 embryos each. Multiple endpoints were assessed including mortality, malformations, minimum concentration to inhibit growth (MCIG), and the teratogenic index (TI). Mortality and malformation effects occurred at different ranges, and therefore, the exposures were conducted in low dose and high dose ranges. The high dose range for mortality effects was conducted at 0, 10, 20, 40, 80, and 160 mg/L of V. The low dose exposures to assess malformation effects were conducted at 0.0001, 0.00025, 0.0005, 0.00075, and 0.001 mg/L. Binary logistic regression was used to determine the LC50 and EC50 for the two sets of definitive tests. The LC50s were determined to be 46.10 mg/L and 26.91 mg/L for VDH_2_O and 34.50 and 25.25 for VMED for the two breeding pairs respectively. The EC50 was calculated as 0.00053 mg/L and 0.00037 mg/L for VDH2O and 0.00036 mg/L and 0.00017 mg/L for VMED for the two definitive tests respectively. The TI was calculated as 86,981 and 72,729 for VDH2O and 95,833 and 148,526 for VMED. Ultimately, there were severe malformation effects in embryos exposed to low doses of V and V was determined to be a very strong teratogen.

## Introduction

Metal pollution is a global environmental issue, with many metals having toxic effects in high concentrations (Imtiaz et al. 2015). Metals are naturally present in sediments, but increased concentrations are found due to pollution. Metals in sediments are at risk of being mobilised from the sediments by changes in physicochemical conditions in water bodies (Imtiaz et al. 2015). One such metal is vanadium (V), a transitional element with multiple oxidation states (Willsky 1990).

Vanadium is known to occur in low concentrations in unimpacted aquatic systems because some forms of V are water-soluble (Tracey et al. 2007). Many industrial and mining processes use V in some of its forms or release V as a waste product into the environment (Tracey et al. 2007). According to the Global Outlook on Vanadium for 2018, 85% of the global V supply is produced by South Africa, Russia, and China (Shaw 2017), and there is an expected increase of 30% V production in China in the near future.

The Frog Embryo Teratogenesis Assay-*Xenopus* (FETAX) is a standardised method developed to evaluate the toxicity of substances on the developmental stages of *Xenopus* species (ASTM 2012). A FETAX assessment can be conducted on single contaminants in water, as well as complex effluent samples and sediments (ASTM 2012; Bantle 1992). The FETAX method consists of a 4-day acute exposure of stage 8–11 (early blastula) *Xenopus laevis* embryos to the toxicant. The mortalities are recorded daily and after the exposure, the 96-h LC_50_ is determined (endpoint 1). After the exposure is done, the embryos are assessed for malformations based on the Atlas of Abnormalities (Bantle et al. 1991). The EC_50_ is then calculated by Probit analysis with the effect being malformation (endpoint 2). The third endpoint of FETAX is the determination of the Minimum Concentration to Inhibit Growth (MCIG). These endpoints are used to determine whether the test substance is teratogenic (Bantle 1992).

The use of a standard FETAX medium was developed by Dawson and Bantle (1987), to reduce the amount of variation in inter- and intra-laboratory studies to validate FETAX as a standard method. Due to the natural variations in dechlorinated tap water used originally, it was difficult to find reproducible results when testing known teratogens in FETAX. FETAX medium, when compared to other media such as modified Ringers solution, was found to increase growth significantly and also increase the rate of development (Dawson and Bantle 1987). However, standard FETAX testing using FETAX medium does not account for the increased or decreased toxicity that could occur in softer waters. In South Africa, there are some rivers and dams that have very low electrical conductivity and very high purity, especially streams in the Drakensberg region (Dunnink et al. 2016). Therefore, a round of FETAX tests were conducted using distilled water, which has an electrical conductivity profile similar to the aforementioned ecosystems. The aim of this study was to determine the toxicity of V_2_O_5_ on *Xenopus laevis* embryos by means of the standard FETAX method to establish if V_2_O_5_ has teratogenic effects when dissolved in distilled water and in FETAX medium.

## Materials and methods

### Test organism care, husbandry, and breeding

*Xenopus laevis* or the African clawed frog is an allotetraploid amphibian species native to Southern Africa. It is a fully aquatic species that has been well established as a laboratory test organism (Cannatella and De Sa 1993; Xenbase 2018). For the purposes of this study, sexually mature adult *Xenopus laevis* males and females were housed in 1000-L standard glass tanks, in controlled environmental rooms. Each tank housed 10 frogs. Adults were kept under the conditions stipulated by the South African National Bureau of Standards for the care and use of animals for scientific purposes (SANS 2008).

The environmental room temperature was set at 22 ± 2 °C with minor fluctuations throughout the study period. The photoperiod for the adults was kept at a standard 12/12-h light/dark cycle. Distilled water was used for the replacements to ensure that other contaminants did not affect the results of the exposure study in the F1 generation.

The breeding of the adults for the purposes of FETAX was conducted as stipulated by the standard FETAX method (ASTM 2012). Two breeding pairs were chosen at random and placed in separate 50-L breeding tanks. The breeding pairs were allowed to acclimate for 24 h before breeding was induced. Males and females were injected into the dorsal sac with human chorionic gonadotropin (HCG). Males were injected with 150 IU and females were injected with 300 IU of HCG as stipulated by the FETAX method (ASTM 2012). The males and females were then placed together overnight for amplexus to commence during the dark cycle. The breeding process was conducted in tanks that were filled with borehole water. The sorting of the embryos for exposure was conducted in a controlled environmental room (temperature: 22 °C ± 2 °C; photoperiod: 12/12).

### Water quality and toxicant

Vanadium pentoxide (V_2_O_5_) (Merck) was chosen for the exposure. It is a very fine (5 µm particle size) orange powder which is dissolvable in water. The motivation for choosing V_2_O_5_ as the toxicant for this study was the known toxicity and previous studies conducted on human, rodent, and aquatic biota toxicity (ATSDR 1992). The V_2_O_5_ was dissolved in both distilled water as well as standard FETAX medium. Concentrations of V in the exposure mediums were tested with the Merck Pharo 100 Spectroquant. The method of determining V concentrations in exposure fluids was a standard method supplied by Merck for the Pharo 100 Spectroquant. The samples were analysed at 405-nm wavelength and a slope of 0.2188. The standard method of determining V concentrations in water supplied by Merck was unsuccessful in determining V concentrations dissolved in FETAX medium. It was however successful in determining V concentrations in both distilled and deionised Milli-Q water. The exposure of *X. laevis* embryos was conducted with various concentrations of V_2_O_5_ dissolved in distilled water as well as FETAX medium. The experiments were conducted at the same time with the same experimental conditions.

### Frog Embryo Teratogenesis Assay – *Xenopus* (FETAX)

#### De-jellying

Eggs were removed from the breeding tanks at random and stored in tank water until reaching stage 8 of development (ASTM 2012). The eggs were cleared of their jelly coating by adding 2% l-cysteine (dissolved in distilled water and pH adjusted to 8.1 with 1-M NaOH). The embryos remained in the l-cysteine solution for no longer than 3 min or until all the jelly was removed (ASTM 2012). The de-jellying process was conducted under a Zeiss Stereoscopic Dissection Microscope to ensure the complete removal of the jelly coating. After treatment with 2% l-cysteine, the embryos were rinsed with distilled water and FETAX medium respectively to remove the remaining l-cysteine and to prevent degeneration of the embryos (ASTM 2012).

#### Embryo selection

Embryos selected for the exposure varied between developmental stages 8–11, determined by the staging guide developed by Niewkoop and Faber (1994). The selection of embryos was based on developmental characteristics for a healthy embryo such as normal cleavage, normal pigmentation, and normal embryo shape as specified by the Atlas of Abnormalities (Bantle et al. 1991). Eggs that were necrotic or laid in strings were not used for the exposure. For each exposure concentration, two replicate dishes were included, containing 15 embryos each.

#### Exposure procedure

At the onset of the exposure, V concentrations and pH were measured in the various exposure fluids. For the static exposure, 35-mm disposable Petri dishes were used. The Petri dishes were inspected every 24 h for mortalities and the dead embryos were removed. Mortality was determined by assessing whether the embryo was responsive to stimulation and whether a heartbeat could be seen under the microscope. Exposure fluid was renewed with fresh aerated exposure fluid every 24 h once the dead embryos were removed. The positions of the Petri dishes containing the embryos were randomly rotated every 24 h.

At the end of the 96-h exposure, the developmental stage of the remaining live embryos was determined. All embryos had reached stage 46 of development. At the end of the exposure, the embryos were placed in 3% formalin solution for further examination. The embryos were assessed for malformations based on the standard FETAX method. The assessment of malformations is based on a ‘yes or no’ system coded in a binary format (0 and 1) but does not account for the severities of the malformations found. The LC_50_ and EC_50_ were calculated using binary logistic regression using SPSS v. 25 and the teratogenic index (TI) was calculated from the results using the following formula:

$$\mathrm{TI}={\mathrm{LC}}_{5}/{\mathrm{EC}}_{50}$$

If the teratogenic index value exceeds 1.3, the substance being tested is considered a teratogen. Teratogens can be classified into the categories based on their TI score (Dawson and Bantle 1987). A TI score of 1.3–2.0 is considered a weak teratogen, 2.0–3.0 a moderate teratogen, and > 3.0 a strong teratogen (Dawson and Bantle 1987).

The MCIG is suggested to be determined as the first concentration to significantly decrease the size of the embryos by means of a 1-sided independent sample’s *t*-test as specified by the standard FETAX protocol (ASTM 2012). Tadpoles were measured under the dissection microscope using digital callipers. The independent sample’s *t*-test was not used in this experiment to determine the MCIG due to the nested experimental design. The data were analysed for significant differences (*P* < 0.05) using the repeated measures ANOVA or nested ANOVA. The analyses were conducted using SPSS v. 25. The assumptions for a nested ANOVA include that the data adhere to sphericity and equal variances (Field 2009). Sphericity is normally tested by Mauchly’s test for sphericity; however, sphericity is only tested when there are more than 3 levels in the data. In this test, there were only 2 levels and so sphericity was assumed (Field 2009). The data were also tested for normality and homogeneity of variance with Levene’s test and the Kolmogorov–Smirnov test and it was determined that the data did not adhere to these assumptions for a nested ANOVA; therefore, the growth inhibition data were log-transformed for the analysis (Field 2009).

#### Range-finding test

A range-finding test was initially conducted to determine the range of effects for V_2_O_5_. This consisted of a series of exposures where 15 embryos were exposed in duplicate to a wide range of V_2_O_5_ concentrations. The concentrations used in the range-finding experiment were 0.001 mg/L, 0.01 mg/L, 0.1 mg/L, 1 mg/L, 10 mg/L, and 100 mg/L (ASTM 2012). The range-finding test was conducted with V_2_O_5_ dissolved in both distilled water (VDH2O) and FETAX medium (VMED). From the range-finding test, the ranges of mortality and malformation concentrations were visually inferred.

#### Definitive tests

The range-finding tests indicated that the effects of V on *X. laevis* embryos possibly occurred at concentrations lower than those included in the initial range-finding tests. It also indicated that the lethal effects of V occurred around the 10–100 mg/L range; therefore, two sets of definitive tests were conducted for V that included a low dose range and a high dose range of V concentrations. The low dose test was conducted with sub-ppb level exposures (0.0001 mg/L, 0.00025 mg/L, 0.0005 mg/L, 0.00075 mg/L, and 0.001 mg/L) to account for the lower range at which malformation effects were observed in the range-finding test. The high dose range was from 10 to 160 mg/L increasing by a factor of 2 to include the range of concentrations in which mortality effects were observed. For each exposure concentration and medium type, two definitive tests were done using embryos from two different breeding pairs to account for possible genetic variation. All definitive test 1 (DT1) tests in the experiment, including low dose and high dose concentrations and medium types, were conducted with embryos from the same breeding pair, and all definitive test 2 (DT2) tests were conducted with embryos from a second breeding pair to allow for direct comparison between the various concentrations and medium types. For each definitive test (1 and 2) and each exposure concentration, two Petri dishes containing 15 embryos each were used. For example, DT1 had two replicate dishes of each concentration containing 15 embryos each. All the tests were conducted at the same time under the same experimental conditions.

## Results

The concentrations of V were measured in the distilled water range-finding test only, by means of spectrophotometry. All were within 80–120% of the nominal concentrations, except for the 1 mg/L group. Concentrations could not be measured in the lower exposure fluids due to the instrument’s range of detection (0.1–5 mg/L). The method of determining the V concentrations was also not successful in the VMED exposures; it is suspected that there were interactions with the ions in the FETAX medium which affected the chemical process. As a result of not having measured concentrations for all of the exposures, the statistical analysis was conducted using nominal concentrations.

### Standard FETAX assessment

#### Range-finding test for VDH_2_O

**Fig. 1:**
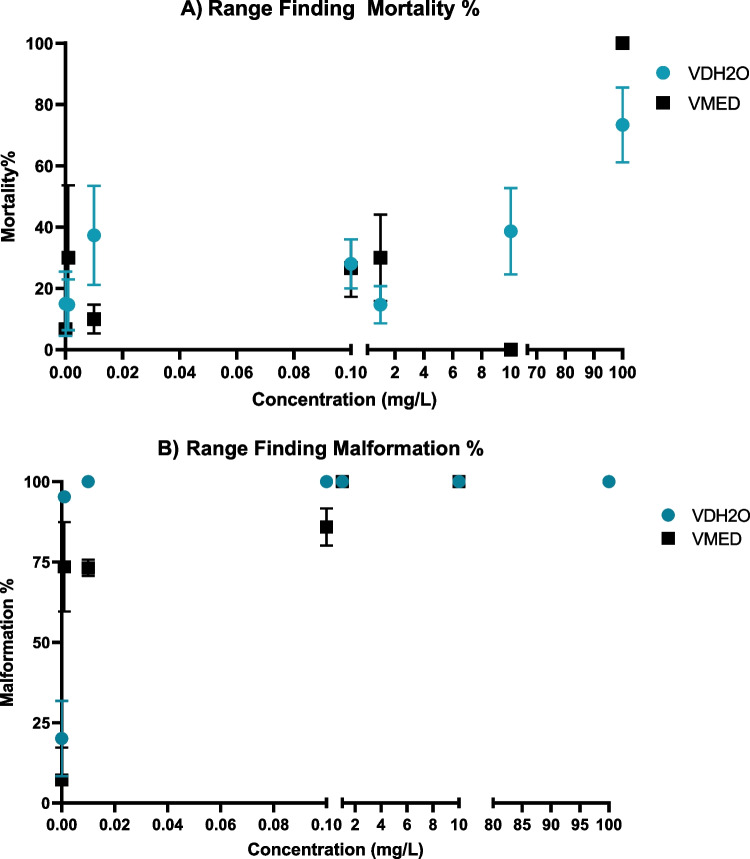
Mortality and malformation percentages found in the 96-h range-finding experiment using vanadium in distilled water (VDH2O) and FETAX medium

The results of the VDH_2_O range-finding test indicated that there is an increase in mortality with an increase in the concentration of V (Fig. 1A). It was identified that the range for mortality effects where the LC_50_ could occur was between 10 and 100 mg/L. Mortalities ranged from 14.28% in the control group to 73.33% in the 100 mg/L group.

The malformations assessed in the range-finding experiment showed a sharp increase in malformed embryos from the lowest exposure concentration (0.001 mg/L) (Fig. 1 B). From the 0.1 to the 100 mg/L groups, 100% of the embryos were malformed. In the control group, 26.67% of the embryos were malformed, but these were mostly only slight alterations in the normal phenotype. As this was only a range-finding test, the higher control effects were noted and reassessed in the definitive tests.

#### Range-finding test for VMED

In the VMED range-finding exposure, there was an observed severe mortality effect in the 10–100 mg/L range (Fig. 1). The control group had a 6.67% mortality rate, followed by increased mortality in the mid-range concentrations. In the 10 mg/L group, no mortalities were observed, whereas in the 100 mg/L group, there were no surviving embryos.

The malformation effects in the VMED exposure were observed to increase sharply between the control and 0.001 mg/L groups, and further increased to 90.48% at 0.1 mg/L and reached 100% in the 1 mg/L and 10 mg/L groups. In the control group, the percentage of malformed embryos remained below the 10% threshold.

#### Definitive tests

**Fig. 2:**
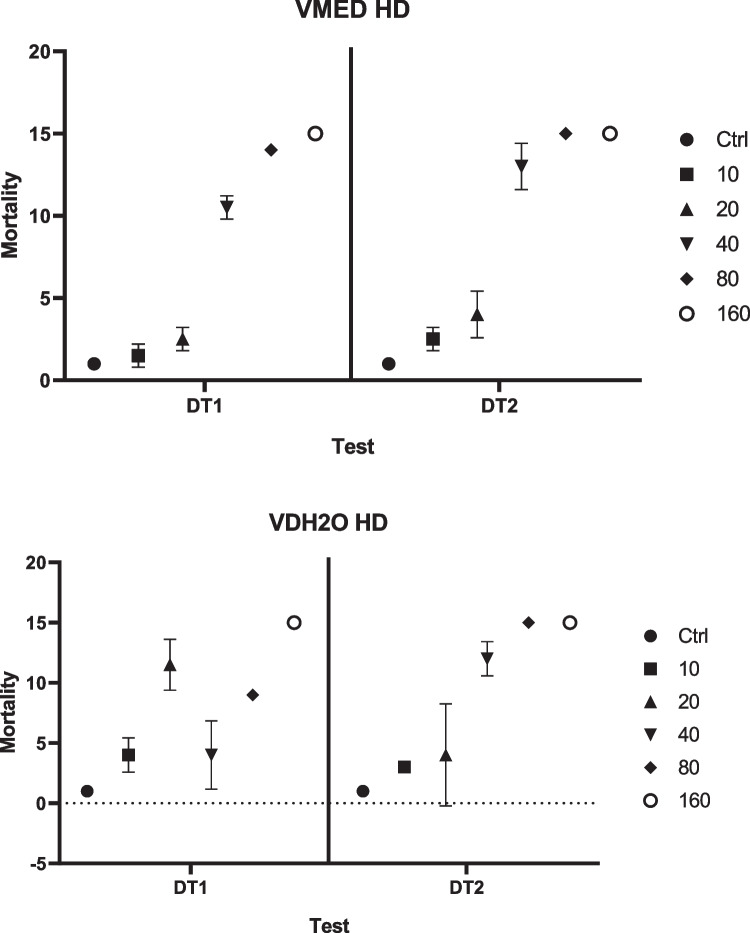
Mortality found in the definitive tests where A represents the mortality observed in the two definitive tests, DT1 and DT2, for vanadium in distilled water (VDH_2_O) as well as vanadium in FETAX medium (VMED). Error bars indicate standard deviation (SD) (*n* = 15)

Figure 2 indicates the mortality effects in the VMED exposures for DT1 and DT2 separately. It is clear that there was little variation in mortality effects between the two definitive tests as well as the replicates. Minor differences can be observed between the mortality effects in DT1 between the two media types, VDH_2_O and VMED. The mortality effects were further investigated in the following sections regarding the determination of LC_50_ concentrations. The high dose VDH_2_O exposures for each of the definitive tests indicated a small amount of variation between the tests but the increasing trend in mortality stayed the same in both DT1 and DT2. Mortality in the control group was observed to occur in 6.67% of cases for both VDH_2_O definitive tests.

#### Growth inhibition

**Fig. 3:**
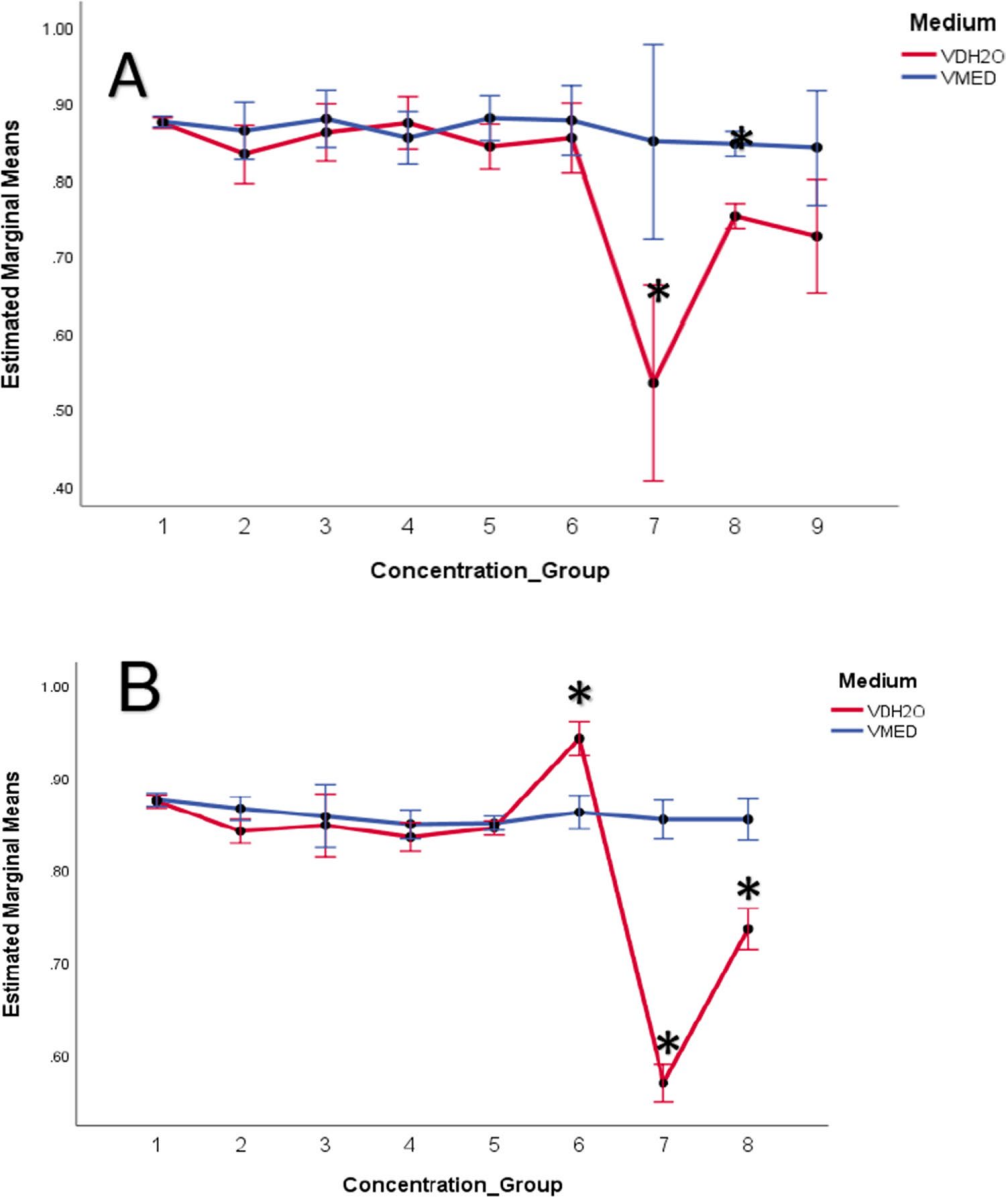
Estimated marginal means of tadpole length in definitive test 1 (**A**) and definitive test 2 (**B**) determined in the two medium types (VDH_2_O and VMED) using a repeated measures ANOVA. Concentration groups include the following: 1 = control, 2 = 0.00025, 3 = 0.0005, 4 = 0.00075, 5 = 0.001, 6 = 10, 7 = 20, 8 = 40, 9 = 80 mg/L. Significant differences from the control are indicated with asterisks (*P* < 0.05). Error bars indicate standard error

The repeated measures ANOVA revealed that there was more severe growth inhibition in the VDH_2_O exposures compared to the VMED, with significant differences detected between the mediums for 20 mg/L in DT1 (*P* = 0.015) and for 10 mg/L (*P* = 0.025), 20 mg/L (*P* = 0.003), and 40 mg/L (*P* = 0.017) in the DT2 exposure (Fig. 3A). In the DT1 exposure, a significant reduction in growth was seen in the tadpoles exposed to VDH_2_O in 20 mg/L (*P* = 0.031) and 40 mg/L (*P* = 0.003) V_2_O_5_. In the VMED DT1 exposure, the first and only significant growth inhibition was detected in the 40 mg/L group (*P* = 0.041).

In the DT2 exposure (Fig. 3B), no significant growth inhibition was detected in the VMED exposure; however, in the VDH_2_O exposure, significant growth inhibition was observed at 0.00025 mg/L (*P* = 0.008), 20 mg/L (*P* = 0.001), and 40 mg/L (*P* = 0.003). According to the FETAX standard guide, the MCIG is the first concentration to inhibit growth significantly, in this test; according to this definition, the results would be inconclusive, with massive variation in the MCIG between tests and mediums. It is however clear from the results of the tests that there was an inhibition of growth in the higher concentrations for VDH_2_O in both definitive tests, and a lesser effect in the VMED exposures.

#### Lethal and effect concentrations, and teratogenic index

**Fig. 4:**
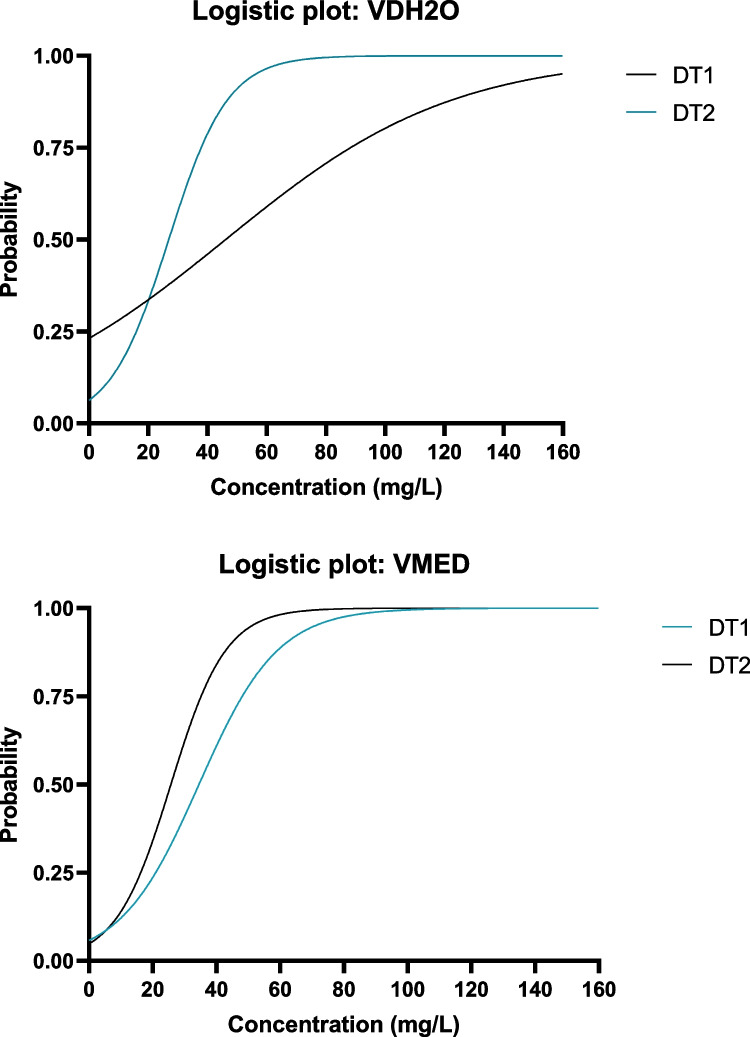
Probability curves determined by binary logistic regression analysis conducted on the mortality data acquired after 96-h exposure to vanadium in distilled water (VDH_2_O) and vanadium in FETAX medium (VMED)

The calculated probability scores from the binary logistic regression analysis indicate that the two definitive tests were similar in terms of mortality in the same medium type (Fig. 4). The graphs plot the probability that an embryo may be dead at a certain concentration based on the exposures conducted. The model fit information for the various binary logistic regressions performed on the mortality data can be seen in Table 1. The binary logistic regression analysis revealed that the LC_50_ values calculated for the VDH_2_O exposures were 46.10 mg/L and 26.91 mg/L respectively for the DT1 and DT2 tests. This is a large difference in lethal concentration between the two different genetic lines. For the VMED exposures, the LC_50_ values were calculated as 34.5 mg/L and 25.25 mg/L respectively for DT1 and DT2. There is also a large concentration difference between the two genetic lines for VMED. In both the VDH_2_O and VMED exposures, the DT1 line was observed to be more resistant to mortality than the DT2 line. When comparing the two medium types in terms of mortality, the LC_50_s of DT1 differed to a greater extent between the two medium types (46.10 and 34.50 mg/L) compared to the LC_50_s of the DT2 line between the two medium types (26.91 and 25.25 mg/L) for VDH_2_O and VMED respectively (Table 3).

**Table 1:** Model fit information for the binary logistic regressions conducted to determine mortality effects observed in embryos exposed to vanadium in distilled water (VDH_2_O) and vanadium in FETAX medium (VMED) for two definitive tests (DT1 and DT2)

		Best-fit values		Std error	Odds ratios	*P*	Goodness of fit	
		β0	X at 50%	β0	β0		Tjur’s *R* squared	Cox-Snell’s *R* squared
VDH_2_O	DT1	− 1.201	46.1	0.243	0.301	< 0.0001	0.253	0.258
	DT2	− 2.703	26.9	0.439	0.067	< 0.0001	0.606	0.531
VMED	DT1	− 2.79	34.5	0.427	0.061	< 0.0001	0.608	0.526
	DT2	− 2.98	25.7	0.481	0.051	< 0.0001	0.646	0.554

**Fig. 5:**
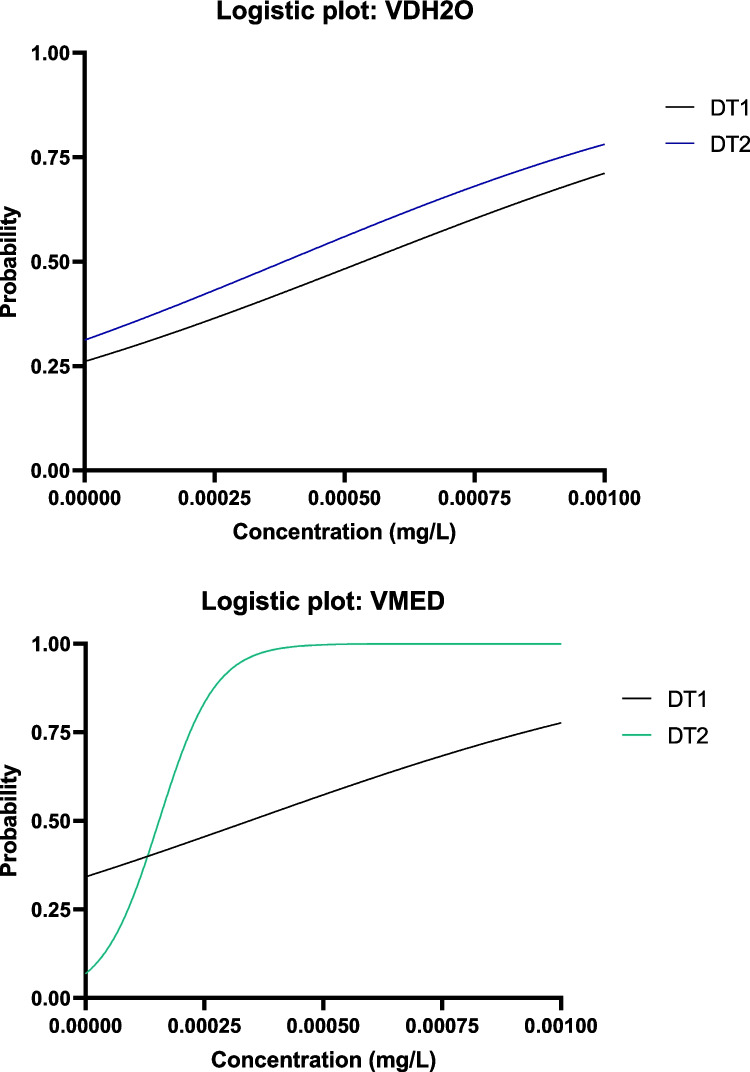
Probability curves determined by binary logistic regression analysis conducted on the malformation data acquired after 96-h exposure to vanadium in distilled water (VDH_2_O) and vanadium in FETAX medium (VMED)

For the EC_50_ determination in VDH_2_O, the two definitive tests had similar slopes yet had slight variation in intercept (Fig. 5). There were two different responses observed in the VMED EC_50_ determinations. In DT1, a plateau was not reached due to none of the concentrations having 100% malformation rate. However, in DT2, a plateau was reached in some of the lower concentrations indicating that there was some variation in malformation response between the two breeding pairs in VMED, and that the embryos from the DT1 genetic line may have not been as sensitive to phenotypic alterations as the embryos from the DT2 genetic line.

The EC_50_s calculated for the various medium types and definitive tests indicate that there were differences between all the variables (Table 2). For the VDH_2_O exposure, the EC_50_s were calculated as 0.00053 mg/L and 0.00035 mg/L respectively for DT1 and DT2. The EC_50_s calculated for the VMED exposure were 0.00036 mg/L and 0.00015 mg/L respectively for DT1 and DT2. The same trend is seen as in mortality effects with a higher resistance to the effects of V observed in the DT1 line compared to the DT2 line.

**Table 2:** Model fit information for the binary logistic regressions conducted to determine malformation effects observed in embryos exposed to vanadium in distilled water (VDH_2_O) and vanadium in FETAX medium (VMED) for two definitive tests (DT1 and DT2)

		Best-fit values		Std error	Odds ratios	*P*	Goodness of fit	
		β0	X at 50%	β0	β0		Tjur’s *R* squared	Cox-Snell’s *R* squared
VDH_2_O	DT	− 1.042	0.000536	0.3258	0.3527	0.0001	0.1056	0.1051
	DT2	− 0.7907	0.000384	0.3152	0.4535	< 0.0001	0.1171	0.1142
VMED	DT1	− 0.6539	0.000344	0.3131	0.52	0.0003	0.1026	0.0979
	DT2	− 2.607	0.000155	0.7323	0.07376	< 0.0001	0.7519	0.5363

Due to the vast difference between the mortality and effect ranges and the resulting differences between the LC_50_s and EC_50_s for the same groups, the TI values calculated for all of the groups indicated that V_2_O_5_ is a strong teratogen (Table 3). The highest TI value was calculated for DT2 exposed to VMED (160,625), followed by DT1 in VMED (101,471), DT1 in VDH_2_O, and finally DT2 in VDH_2_O (85,370). The results suggest that there may be a stronger toxic effect in VMED compared to VDH_2_O that requires further analysis.

**Table 3:** LC_50_, EC_50_, and teratogenic index (TI) values calculated for the embryos exposed to vanadium in distilled water (VDH_2_O) and vanadium in FETAX medium (VMED) for two definitive tests (DT1 and DT2)

Medium	Test	LC_50_	EC_50_	TI
VDH_2_O	DT1 DT2	46.1 26.9	0.00054 0.00038	85,370 70,789
VMED	DT1 DT2	34.5 25.7	0.00034 0.00016	101,471 160,625

**Fig. 6:**
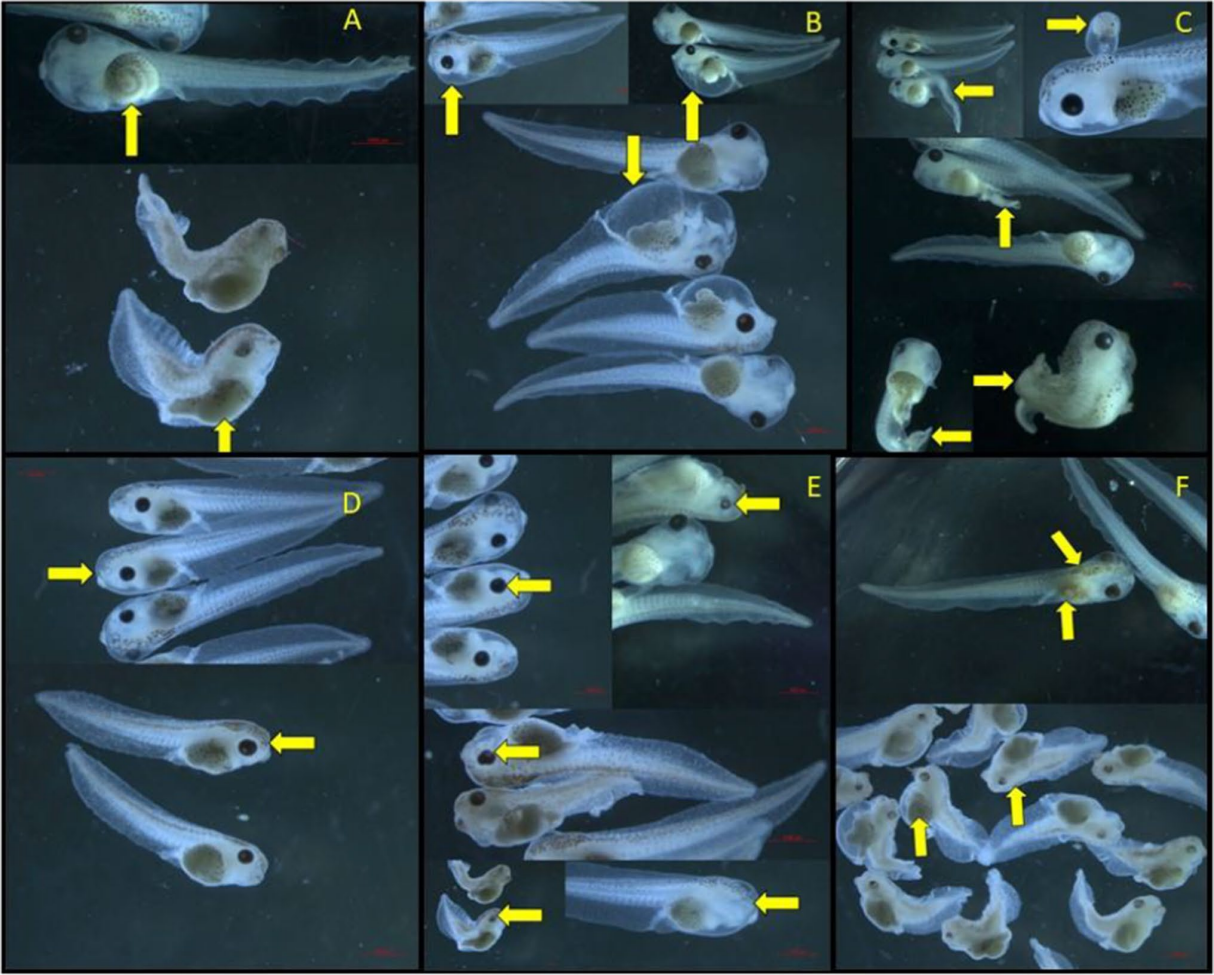
**A** Normal gut (top) and severely uncoiled gut (bottom). **B** Various forms and severities of edema. **C**) Axial malformations including axial bending, brainstem growth, multiple tails. **D** Normal head size (top) as opposed to severe microcephaly (bottom). **E** Normal eyes (top left) compared to reduced eye size (top right), burst cornea (middle), blindness (bottom left), and no eye development (bottom right). **F** Hyperpigmentation (top) of the gut and brainstem as well as severe hypopigmentation of the same areas (bottom)

The exposed embryos presented with a variety of malformations ranging from mild to severe in the various concentrations. The most prevalent gut malformation observed was gut uncoiling in various stages. In some instances, the gut had no coiling and was a straight tube extending from the mouth to anus. Figure 6A indicates normal gut coiling compared to uncoiling. In terms of various types of edema, it was observed that there were embryos that presented with facial, cardiac, and abdominal edema in many instances, but that cephalic and optic edema was rarely induced (Fig. 6B). It was also observed that the exposed embryos presented with various axial-related deformities of the tail, fin, and notochord (Fig. 6C). Axial bending and shortening were some of the more prevalent axial malformations noted. Thinning of the fins was also observed in many cases of exposed embryos. In some severe cases, embryos developed with two heavily deformed tails. There was one instance where an embryo developed a growth from the brainstem which seemed to contain gut tissue (Fig. 6C). Facial malformations were common in the exposed embryos. The most notable of the facial malformations was microcephaly — a reduced head size (Fig. 6D). Malformations of the eye were present in various forms and severities. Eye malformations included burst corneas, eye sizes reduced or enlarged, and even no development of the eyes at all. In some of the high concentrations, the embryos were all completely blind (Fig. 6E). In terms of pigmentation, there were many instances of various levels of hyperpigmentation, especially in the gut and brainstem. Hypopigmentation was seen mostly in the most severe cases of malformation (Fig. 6F).

## Discussion

The results from this study revealed that V_2_O_5_ is a strong teratogenic substance with a severe risk of inducing embryonic defects. There was a large difference between the range of concentrations that induced malformations and the range of concentrations which caused mortalities, with the EC_50_ concentrations being lower than 1 µg/L and the LC_50_ concentrations ranging between 25.25 and 46.10 mg/L in the two exposure mediums and two different breeding pairs. The TI scores calculated for V_2_O_5_ were all exceptionally high compared to the TI scores of other known teratogenic substances. For instance, V was notably higher than Cu (TI = 8.8) and Zn (TI = 21) (Luo et al. 1993) and higher than roundup which had a calculated TI of 3.4 (Bonfanti et al. 2018). Nifidipine, a pharmaceutical agent, was determined to have a TI of 101 (Boğa Pekmezekmek et al. 2015). During the interlaboratory validation studies of the FETAX method, the TI values of 5-fluorouracil (TI = 6.7–18.7), caffeine (TI = 1.8–3.4), isoniazid (TI = 4.1–72.8), and 6-aminonicotinaminde (TI = 241.6 620.0) were assessed for variability (Bantle et al. 1994a, b). The variability is attributed to the subjective nature of malformation assessments. According to the standard FETAX method, it is possible for the TI score to range into the thousands with strong teratogenic substances (ASTM 2012).

The 96-h LC_50_ concentration calculated was notably higher than the 24-h LC_50_ for V in *Artemia* species which presented LC_50_ values of 0.0107 mg/L and 0.011 mg/L (Baniamam 2014). Other available mortality data on V in aquatic environments include 96-h LC_50_ values on guppies *Poecilia reticulata* (0.0102 mg/L), zebrafish *Danio rerio* (5.3 mg/L), carp *Cyprinus carpio* (32.4 mg/L), and rainbow trout *Oncorhynchus mykiss* (11.8 mg/L) (Ohio EPA 2006). This indicates that the *X. laevis* embryos are more resistant to V in aquatic environments than multiple other aquatic species in terms of mortality but have a similar resistance to *C. carpio*. It is, however, pertinent to note that the malformations induced by the V exposure affected the *Xenopus* embryos at similar and lower concentrations that induced mortality in other aquatic species and some of these malformations may compromise the survival of the tadpoles in natural systems due to their inability to feed, swim, and avoid predation.

In this study, the growth of the tadpoles that were exposed to FETAX medium was not as affected in either of the breeding pairs used, compared to the tadpoles exposed to distilled water. There were no differences in the controls or most of the lower exposure concentrations between the medium types; the effects of growth inhibition were seen mainly in the high concentrations of the tadpoles that were exposed to distilled water. This indicates that V possibly has a stronger growth inhibition effect in softer water with fewer competing ions, or that the stress of low concentrations of ions is affecting the growth of the tadpoles in the presence of vanadium more.

According to a review paper by Imtiaz et al. (2015), the concentrations of V in freshwater systems have been found to occur up to 0.0645 mg/L. A study conducted on a hot water spring in Brazil used for its medicinal properties revealed that the levels of V in the water ranged from 0.00022 to 0.829 mg/L (Trovo et al. 2017). Other studies revealed concentrations of V in freshwater systems in the Nyl River, South Africa (0.009 mg/L) (Baker 2018).

The current study has shown that there are significant effects observed at these environmentally relevant concentrations such as significant malformation incidence, including edema, gut uncoiling, fin narrowing, and microcephaly.

The exposure of *X. laevis* embryos to V has indicated that although the embryos may be affected by malformations, which may ultimately hinder their chances of surviving to adulthood, V does not induce serious mortality at environmentally relevant concentrations. It should also be noted that these exposures were conducted acutely and that some more serious effects may be found in chronic exposure to low concentrations of V. It was recently determined that adult *X. laevis* exposed to V had significant physiological responses after acute and chronic exposures (Dahms-Verster et al. 2020).

According to the standard FETAX method, as well as various other studies (ASTM 2012; Bantle 1992), the results from FETAX may be extrapolated to mammals, and as such, the risk to humans may be tentatively deduced from FETAX at a 75% accuracy level. This could be increased to an 85% accuracy level by incorporating the metabolic activation system (ASTM 2012; Bantle 1992). Therefore, further studies are required to increase the accuracy level of the teratogenic effects of V in FETAX so that a better understanding can be acquired about the possible risk to developing human embryos. Further assessment is also advised with the use of the enhanced FETAX method developed by Hu et al. (2015) where a severity score is assigned to each malformation. This gives more detailed insight into the malformations caused by the exposure.

## Conclusion

The concentrations of V in aquatic ecosystems may increase due to various forms of pollution. The exposure of *X. laevis* embryos to various ranges of concentrations revealed that this metal is indeed a teratogenic substance which could cause malformations during development at environmentally relevant concentrations. The effect on the tadpoles in terms of V-induced mortality was not nearly as high as the induction of malformations by the V, but this is not to say that the induced malformations may not hinder the survival of the tadpoles to adulthood.

In this study, it was determined that growth inhibition was more severe overall in the tadpoles in the distilled water medium; however, mortality and malformation incidence was more severe in the FETAX medium. This study also revealed that ion concentrations in an aquatic system may increase the toxicity of V based on the number of competing molecules in the water. It was also established that the one breeding pair used in the experiment was consistently more sensitive to the V exposure in terms of mortality and malformation effects, indicating that there may be a genetic factor to the resistance of the tadpoles to V toxicity.

It is recommended that other forms of V, such as sodium orthovanadate, be tested by FETAX and compared to V_2_O_5_. An in-depth study of the pathways of bioaccumulation may also be a valuable avenue to take in terms of future studies. Ultimately, a better understanding of the risk and toxicity of V in both aquatic systems, as well as in mammalian development, is the goal for future research.

## Data Availability

Data is available from the corresponding author on request.
